# Fundamentals of deep brain stimulation for Parkinson's disease in clinical practice: part 1

**DOI:** 10.1055/s-0044-1786026

**Published:** 2024-04-23

**Authors:** Camila Henriques de Aquino, Mariana Moscovich, Murilo Martinez Marinho, Lorena Broseghini Barcelos, André C. Felício, Matthew Halverson, Clement Hamani, Henrique Ballalai Ferraz, Renato Puppi Munhoz

**Affiliations:** 1 University of Calgary, Cumming School of Medicine, Department of Clinical Neurosciences, Calgary, AB, Canada.; 2 University of Calgary, Hotchkiss Brain Institute, Calgary, AB, Canada.; 3 Universidade Federal de São Paulo, Escola Paulista de Medicina, Departamento de Neurologia e Neurocirurgia, São Paulo SP, Brazil.; 4 Christian-Albrechts University, Department of Neurology, Kiel, Germany.; 5 Hospital Israelita Albert Einstein, São Paulo SP, Brazil.; 6 University of Utah, Department of Neurology, Salt Lake City, Utah, United States.; 7 University of Toronto, Sunnybrook Hospital, Toronto, ON, Canada.; 8 University of Toronto, Toronto Western Hospital, Toronto, ON, Canada.

**Keywords:** Deep Brain Stimulation, Parkinson Disease, Neurosurgery, Estimulação Encefálica Profunda, Doença de Parkinson, Neurocirurgia

## Abstract

Deep brain stimulation (DBS) is recognized as an established therapy for Parkinson's disease (PD) and other movement disorders in the light of the developments seen over the past three decades. Long-term efficacy is established for PD with documented improvement in the cardinal motor symptoms of PD and levodopa-induced complications, such as motor fluctuations and dyskinesias. Timing of patient selection is crucial to obtain optimal benefits from DBS therapy, before PD complications become irreversible. The objective of this first part review is to examine the fundamental concepts of DBS for PD in clinical practice, discussing the historical aspects, patient selection, potential effects of DBS on motor and non-motor symptoms, and the practical management of patients after surgery.

## Resumo

Nas últimas três décadas, a estimulação cerebral profunda (ECP) se tornou um tratamento bem estabelecido para doença de Parkinson (DP) e outros transtornos do movimento. A eficácia a longo prazo na DP foi bem documentada para a melhora dos sintomas motores cardinais da DP e das complicações induzidas pelo uso do levodopa, como as flutuações motoras e as discinesias. O momento da seleção do paciente é crucial para se obter os benefícios ideais da ECP, antes que as complicações da DP se tornem irreversíveis. O objetivo desta primeira parte da revisão é examinar os conceitos fundamentais da ECP na prática clínica, discutindo os aspectos históricos, a seleção de pacientes, os potenciais efeitos da ECP nos sintomas motores e não motores da doença e o manejo prático dos pacientes após a cirurgia.

## INTRODUCTION

Although the history of deep brain stimulation (DBS) in movement disorders started with the concept of stereotaxic in the early 20th century, several decades passed until the first stereotactic frame designed for humans was developed in the late 1940s. Interventions in the basal ganglia (BG) started to be explored at that time and were further reinforced after the observation of improvement of tremor in a patient with Parkinson's disease (PD) after the accidental ligation of the anterior choroidal artery. 1 2 The first approach was lesioning the inner segment of the internal globus pallidus (GPi) and the fields of Forel, which, 3 despite the good outcome for rigidity, induced variable improvements for tremor. Afterward, pallidotomy was gradually replaced by thalamotomy, resulting in better tremor control. 4 With the introduction of levodopa in the 1960s, stereotactic surgery became less popular and did not re-emerge until the early 1990s when the shortcomings of levodopa therapy, including levodopa-induced dyskinesias (LID) and motor fluctuations, were fully appreciated. 5 6 7 In addition, advances in physiology, surgery, and neuroimaging played important roles in the revival of stereotactic surgery for PD. Once again, thalamotomy was reintroduced 8 and later the posteroventral GPi became the preferred surgical target, with favorable outcomes not only for the cardinal signs of PD but also dyskinesias. 9 In fact, anatomic and physiological studies suggested that both the GPi and the subthalamic nucleus (STN) were overactive in PD and experimental studies demonstrated that lesions in these structures could improve parkinsonism in animal models. 10 11 12 However, the classical BG model based on the unbalanced activity of the direct and indirect pathways predicted that GPi lesions should worsen dyskinesia, which is not observed in clinical practice. Indeed, the observation of striking benefit on dyskinesia after pallidotomy reinforced the emerging concept of abnormal firing patterns within the BG circuitry as the underlying mechanism of movement disorders. 13

Interventions in the STN were described in 1963, in a series on “diencephalic operations” in 58 patients for the treatment of Parkinsonian tremor that showed a 75% improvement and low incidence of long-term complications. This study also suggested that the posterior STN was the most efficient target, including the fields of Forel, zona incerta, and the prerubral area. 14

The concept of high-frequency stimulation, which would later evolve into DBS, was initially explored in the early 1960s in studies that showed improvement of Parkinsonian tremor with stimulation of the ventrointermediate (VIM) thalamic nuclei at 100–200Hz. 15 Approximately 20 years later, Benabid et al. heralded the modern era of DBS through their series of VIM stimulations in patients with tremors and previous contralateral thalamotomy 16 leading to refinement of the technique over the following years. 17 The first reports of STN DBS in patients with PD were published by the same pioneers in 1993, showing an improvement of 42–84% in motor scores. 18 In 1994, a group from Switzerland also published the first results of three cases of PD treated with pallidal DBS, demonstrating good outcomes regarding motor signs of parkinsonism and LID. 19 The debate around STN versus GPI remains to this day and will be discussed in this review.

## REASONS FOR REFERRING A PD PATIENT FOR DBS

A phenomenon known as “honeymoon period” has been used to describe the prolonged and sustained benefit of levodopa therapy in the early stages of PD. However, over time, patients tend to develop motor complications, such as motor fluctuations and LID. 20 These symptoms emerge at a range of 10% of PD patients per year, 21 affecting virtually all patients in long-term follow-up. 22

Motor fluctuations manifest with a shortening of the therapeutic effect, which leads to the recurrence of PD motor symptoms before the next scheduled dose. With disease progression, PD symptoms tend to cycle more rapidly leading to a sharp loss of therapeutic benefit often designated as “unpredictable off” or sudden on/off phenomenon. 20 Such fluctuations can be accompanied by non-motor symptoms, i.e., psychiatric, autonomic, and sensory complaints. 23 The most intuitive approach to fluctuations' management is the adjustment of levodopa timing at shorter intervals, followed by the addition of different drug classes such as catechol-O-methyltransferase inhibitors, dopamine agonists, or monoamine oxidase B inhibitors. 24 These strategies may be successful for a limited time as the disease continues to progress leading to more complex medication regimens. 25

LID presents with a combination of random choreiform movements and/or dystonia that occur in a wide range of severity, eventually leading to troublesome symptoms, as patients require higher cumulative doses of levodopa. 25 26

Patients experiencing troublesome levodopa responsive motor symptoms that cannot be adequately addressed by medications due to the co-occurrence of intractable LID or complex motor fluctuations are the ones who benefit from a referral for DBS. 26 The effects of DBS largely mirror the best response to levodopa with the exception of refractory tremor, which despite often being poorly responsive to medical therapy, tends to respond well to stereotactic surgery.

Finally, timing for referring patients for these procedures is crucial to avoid reaching a disability level in which an acceptable quality of life can no longer be rescued by DBS or other forms of advanced therapy.

## SELECTION OF DBS CANDIDATES

### Confirmation of PD diagnosis and other criteria

Even among movement disorder specialists, diagnosis of PD in its early stages can be challenging. For example, 10% misdiagnosis is seen in movement disorder centers 27 and ∼25% in non-specialized centers. 28 Although PD patients are usually referred to DBS at more advanced disease stages when potential red flags for alternative diagnoses are more evident, it is always essential to revisit and ascertain the final diagnosis. 29 In addition to confirming the diagnosis, eligibility based on recognition of inclusion or exclusion criteria for DBS therapy is a laborious, intricate step that should be placed at the core of this assessment. 30 This section will discuss some of the pivotal variables in this process.

### Age and disease duration

Age at the time of surgery is an important but rather imprecise variable used in the selection process for DBS in PD. Various centers tend to use arbitrary cut-offs above which patients should have their indication questioned given the higher incidence of complications and higher rate of surgical complications. In addition, older age per se may be associated with more co-morbidity and presence of disabling non-motor and motor symptoms (cognitive decline, depression, levodopa-resistant symptoms, especially axial). 31 A limit of 65, 70, 75, and 80 years old has been applied by different DBS experts and clinical trials, while the importance of an individual patient's functional status, often independent of the chronological age, is the main parameter considered in many centers. 31 32 33 34 35 As highlighted, these cutoffs are arbitrary, based on limited and often contradictory evidence. For example, in a retrospective study evaluating acute complications in 5464 patients submitted to surgical therapies for movement disorders, ∼75% has been PD, found higher mortality and complication rates in patients older than 70 years old, with age working as a surrogate for more comorbidities and complications. 36 On the other hand, another study evaluating perioperative complications in 1757 PD patients submitted to DBS implants found no increase in hospital length stay or complication rates in patients older than 75 years old compared with younger patients. 32 The later study, however, was limited to a 90-day post-operative assessment, while it is essential to keep in mind that acute complications are not the main concerns when considering older patients for DBS, 37 which typically involves the potential negative impact on cognition and axial motor symptoms and signs.

Despite these uncertainties, most of the literature points to a “the earlier, the better” approach with studies showing that despite improvement in motor complications, there is an overall worsening in the “on” medication scores, activities of daily living, and axial disability in the elderly. 38 Also, older patients submitted to STN DBS are at increased risk for a negative effect on quality of life, particularly concerning mobility, activities of daily living, and cognition. 39 A recent systematic review found that in four studies, older age was associated with lower quality of life improvement in the short-term follow-up, whereas in the other six studies, age was not correlated with postoperative quality of life. 40 Age as a variable for eligibility for DBS is a moving target with no clear cut-off but rather a variable highly influenced by other clinical aspects, such as cognitive status, motor phenotype, levodopa response, and the presence of medical comorbidities. 37

Similarly, age and disease duration seem to be interchangeable variables in this equation keeping in mind that DBS should not be considered as a last resort, especially after the essential change in the concept of adequate timing for surgery over the past few years. This paradigm change occurred mainly due to the recognition that the potential gains observable with DBS may not be as meaningful compared with the cumulative residual deficits that can be part of the disease phenotype after a certain stage in the disease course. In other words, the comparative improvements in motor scores may not be mirrored by gains in functionality and quality of life. 29 This is particularly true when motor axial symptoms (i.e., postural instability and gait and speech disorders) and non-motor signs (cognitive dysfunction and mood and behavior changes) are present. This scenario has, indeed, been described back in 2006 by a landmark study showing that patients who were between 10 to 14 years of PD diagnosis experienced lower than expected subjective satisfaction measured by psychosocial outcomes, despite significant improvements in motor scores assessed by their examiners. 41

Another important study arguing against the use of DBS as a late resource is the EARLYSTIM trial, which enrolled 251 subjects, with a mean disease duration of 7.5 years, mean age of 52 years old, Hoehn & Yahr scale score below 3, and presence of motor complications for ∼1.5 years, to receive bilateral STN or the best medical therapy. 42 The results favored the group receiving STN stimulation, with improved quality of life scores of 26% *vs* . -1% for the group that received the best medical therapy. Also, stimulation was superior concerning motor disability, daily living activities, motor complications, and time with good mobility and no dyskinesia. 42 Importantly, the rate of adverse events was relatively similar between the two groups. 42 Despite these encouraging results it is important to emphasize that as part of the inclusion criteria, patients had more than four years of PD diagnosis and had developed significant motor complications. Obviously, different criteria apply to exceptional circumstances, as in the case of very early interventions for patients with tremor-dominant PD with refractory symptoms. 43

### Responsiveness to levodopa

Pre-operative assessment for DBS has been one of the main indications to perform a levodopa challenge test (LCT), since, to date, the response to levodopa as measured during this standardized test is considered the best predictor of DBS motor outcomes. 44

The LCT typically involves an initial evaluation of patients' motor state at their baseline. In most centers, patients are requested to withdraw from their PD treatments for 12 hours before the assessment, which is considered a practically defined “off medication” state. 45 It is common for centers to schedule the LCT in the morning, so patients can withhold their medications during the night, and present to the clinic in the morning, before their first daily dose. It is important to keep in mind that for some patients, off dystonia occurring during the night, or severe morning akinesia, may limit their ability to tolerate a 12-hour PD treatment withdrawal, therefore, an individualized approach may need to be considered for those cases. 46

It is recommended the use of a standardized and validated scale during the LCT, therefore most DBS centers have used the UPDRS or the MDS-UPDRS, applied by an experienced certified practitioner. Neurologists, nurses, physician assistants, and physiotherapists have performed LCT, and in some centers, the assessments are videotaped for future reference during the DBS multidisciplinary meetings, where patients' candidacy and target decisions are reviewed. While most centers focus on the response to the part III (motor) part of the MDS-UPDRS, specific symptoms can also be evaluated with other objective measures, for instance, 3 m or 12 m walk time, timed up and go test, speech and voice analysis, and swallowing evaluation. 46 47 48 49 50

After the baseline assessment in the off state, patients receive a dose of levodopa (combined with dopa decarboxylase inhibitor, such as carbidopa or benserazide). The exact dose prescribed can range from patients' habitual morning dose to a supramaximal dose of 120 to 150% of their habitual morning dose. In some cases, for instance, when patients have drug-resistant tremors or fail to respond in the first LCT, a dose of up to 200% of their morning dose can be recommended. 46

Generally, patients report the onset of the levodopa benefit ∼30 minutes after taking their dose on an empty stomach, with the peak of the dose occurring at 60 minutes. For the purpose of the evaluation of motor response, the peak of the benefit is used to assess the motor symptoms in the “on medication” state; however, assessing the onset of effect and wearing-off phases may also be beneficial in some cases, particularly for evaluation of dyskinesias and duration of response to levodopa. 46

The same motor scale used in the off state is repeated when patients achieve the peak of the levodopa benefit. The percentage of levodopa responsiveness (%LR) is calculated with the formula: %LR = [(off MDS-UPDRS part III score - on MDS-UPDRS part III score) / off MDS-UPDRS part III score] x 100 [ref 2 ]. An improvement of 33% or higher from baseline “off” *versus “* on” test has been considered a predictor of responsiveness to levodopa and consequently accepted as a marker of suitability for DBS. 51 52 During the on-state, it is also recommended to perform the assessment of LID. Several validated rating scales can be applied in the rating of LID, including, the scale proposed by the Core Assessment Program for Intracerebral Transplantation (CAPSIT), 45 the Abnormal Involuntary Movement Scale (AIMS), 53 the Rush dyskinesia rating scale. 54 and the Unified dyskinesia rating scale (UDysRS). 55

It is common good practice to assess patients' vital signs in the off and on states during an LCT. This may allow the identification of autonomic changes and orthostatic hypotension, which may influence the outcomes of the LCT. In addition, non-motor symptoms can be assessed using the non-motor symptoms scale (NMSS), 56 anxiety, depression, and neuropsychiatric scales. Health-related quality of life can also be measured using the Parkinson's disease questionnaire (PDQ-39) 57 or other non-specific quality of life scales.

### Cognitive aspects

Despite the remarkable success of STN DBS in alleviating disabling motor symptoms and improving quality of life, its‘ effects on cognitive functions and its psychiatric co-morbidities are not fully established, even after three decades since the implementation of DBS in clinical practice. 58 59 Various degrees and spectra of cognitive dysfunction are observed in patients with PD, with higher prevalence with advanced age and disease progression. 60 The most affected cognitive domains in PD are executive function, visuospatial processes, and attention. 61 Full-blown dementia suggests a more widespread and dense PD pathology, which not only represents a marker to a less robust motor response to DBS but most of all, a risk for further worsening of cognitive status in the short and long-term follow-up after DBS surgery. 51 62 Additionally, patients with mild cognitive impairment preoperatively appear to be at the highest risk for cognitive deterioration after surgery and should be evaluated individually with caution.

As such, a formal neuropsychological assessment has an essential role in providing an objective profile of cognitive status and confirming eligibility for DBS in PD. As for specific tools for this assessment, it is recommended to include broad measurements of cognitive functioning, such as executive functions (working memory, attention, conceptualization, set activation, set-shifting, and set maintenance), instrumental functions (language, visuo-constructive, visuospatial, visuo-perceptive), and memory. 63

A comprehensive assessment of global cognitive ability in PD can be obtained with the *Mattis Dementia Rating Scale* (MDRS), which is considered one of the appropriate scales to evaluate the loss of global intellectual capacities, especially for subcortical degenerative disease. 64 This scale provides cut-off scores that allow for a psychometric distinction between demented and non-demented patients, however, it may have reduced sensitivity in younger patients being considered for DBS. The MDRS assesses attention, initiation/perseveration, conceptualization, construction, and memory.

The Montreal Cognitive Assessment (MoCA) has been widely used as a screening tool for cognitive dysfunction in PD. Given the availability in 52 languages, and the rapid application (∼10 minutes) it has been used as a preliminary cognitive screening during the evaluation for DBS; however, most centers perform a subsequent, more detailed neuropsychological assessment to better characterize patients’ cognitive profile before DBS surgery. 65

Another scale that can be used for global cognitive assessment is the Parkinson's Disease-Cognitive Rating Scale (PD-CRS). This scale has a good correlation with the MDRS, however with a more rapid administration. 65 When applying cognitive assessment tools in patients with PD, it is important to consider the educational level of the target population, particularly in a diverse country, like Brazil, in which socio-demographic characteristics might influence patients' performance on psychometric tests.

### Psychiatric profile

Neuropsychiatric symptoms and signs are intrinsic aspects of PD and include features of depression, anxiety, apathy, fatigue, and psychosis. From a pathophysiologic standpoint, these symptoms can be a direct result of a neuropathologic process but also can be exacerbated or directly caused by medications used to control motor signs of PD. The effect of DBS on mood is conflicting in the literature with studies suggesting improvement after surgery, while others suggest that depression and anxiety can worsen after the procedure. 66 This correlation can be particularly difficult to disentangle from the effect of changes in medication regimen performed upon programming and the impact of the surgical trauma per se, which can potentially interfere with psychiatric outcome after DBS, particularly of the STN. 66

In this context, patients with unstable mood disorders or at risk for suicide should not be considered for DBS. Although patients with psychotic symptoms often respond to basic therapeutic regimen adjustments, surgery should only be considered once the psychiatric status is considered stable as medication adjustments necessary in the postoperative period might represent another challenge in regards to non-motor symptoms in general, particularly in the case of STN DBS. This recommendation is based on a few different points:

- the envisioned medication reduction should not be as anticipated;
- the procedure carries a psychological burden (withdrawal of dopamine agonists, for example) that should be accounted for;
- changes in basal ganglia physiology induced by DBS may affect the non-motor function of the involved nuclei (i.e., “limbic” STN stimulation); and
- psychosis may be part of the spectrum of incipient cognitive decline not captured by neuropsychological assessment.

In terms of psychiatric assessment tools, the Neuropsychiatric Inventory (NPI), Montgomery and Asberg Depression Rating Scale (MADRS), or a self-rating questionnaire, e.g., Beck Depression Inventory (BDI) have been recommended although, when available, a formal psychiatric consult for the patient and caregiver done by a dedicated professional is preferable. 67

## EFFECTS OF DBS ON PARKINSON'S DISEASE SYMPTOMS

For this review, we discuss the influences of DBS on the most common motor and non-motor symptoms of PD 68 by dividing these effects into those that are:

- consistently beneficial (main indications for surgery);
- neutral, i.e., may or may not occur and, by themselves, are not absolute indications nor contraindications; and
- potentially detrimental (main contraindications).

As a background, it should be kept in mind that the BG functions are not restricted to motor behavior but also concerned with diverse sensory, cognitive, emotional, and autonomic information and, as such, neuromodulation of the structures targeted for motor control should avoid, or minimally interact, with non-motor physiological functioning. 69

### Features that consistently improve with surgery

Rest tremor, rigidity, and bradykinesia have consistent and dramatic responses to either GPi or STN DBS, especially if they are appendicular and levodopa responsive. As already mentioned, the magnitude of levodopa responsiveness correlates directly with surgical outcomes, resulting in improvements in the motor UPDRS with stimulation only (i.e., OFF medication) in the range of 50%, with better results for tremor (around 80%), but also very significant for rigidity and bradykinesia (40 to 60%) at six months postoperatively, remaining stable for up to 6 years. The improvement in the ON medication scores is less robust (15 to 25%), reinforcing the fact that preoperative levodopa responsiveness is essential. Effectiveness seems to be similar for both targets in most studies; however, some have shown a trend to a better outcome for STN DBS patients but fewer adverse events for GPi DBS. 70 71 72 73

Regarding dyskinesia, both GPi and STN DBS demonstrated good outcomes, with improvement in LID scores from 40 to 88% across studies. GPi DBS has a *direct* antidyskinetic effect, 74 while STN DBS may have an indirect effect related to the reduction of dopaminergic drug dosages after surgery. The persistence or worsening of LID after STN DBS is common and, in fact, indicates the necessity to reduce the dose of levodopa. The typical reduction of levodopa equivalent daily doses ranges from 31 to 47%.

Motor fluctuations are also improved by STN and GPi procedures. In a study comparing these targets, the mean increment in time spent in the ON state without LID was ∼4 hours (almost 50%), while the OFF time decreased by 3.5 hours (almost 60%). The changes were virtually the same for both procedures, however, as STN DBS more commonly enables a reduction of medication dosages and number of daily intakes, this technique is favored in cases with more severe motor fluctuations. 73

### Features that may or may not improve with surgery

Gait and postural problems reflect the progression of PD and are often resistant to pharmacological and surgical treatment. The initial reports of the effect of STN and GPi DBS on these parameters were mixed and tended to show modest benefits. One meta-analysis showed that during the first year after surgery, STN DBS improved gait and postural deficits to the same amount induced by medication before the procedure, added by a synergistic effect of medication in the short term. 75 Findings from several studies have shown that freezing episodes that improve with medication may also improve with STN DBS; however, a relatively large proportion of patients show only subtle or no gait and postural improvement after surgery, even during the first year. 76 More refined strategies, such as stimulation using low frequency, may be potentially beneficial in improving certain aspects of gait, including freezing, 77 however, the effect may not be long-lasting, and eventually, these features tend to worsen despite the use of different techniques. The long-term efficacy of GPi DBS is less well documented. As in the case of STN DBS, levodopa may have a synergistic effect on GPi procedures shown in randomized, double-blind studies and one meta-regression review. 78 79

Dysautonomic symptoms are not expected to be changed by either GPi or STN DBS; however, an indirect effect related to improved mobility and, in the specific case of STN DBS, reduction of dopaminergic medication, may lead to positive changes in bowel function, orthostatic hypotension and excessive sweating secondary to dyskinesias. 80

Sleep disturbances are common in PD, affecting 74 to 98% of patients. Several sleep problems may occur, including insomnia, sleep fragmentation, nocturia, nocturnal motor fluctuations, excessive daytime sleepiness, and REM sleep behavior disorder. Although most of the literature does not provide a convincing substrate for a direct effect of DBS, a few studies revealed modest but statistically significant beneficial changes in general subjective sleep quality, a finding potentially indirectly driven by the reduction in anti-Parkinsonian medications and better nocturnal mobility. 81 82 In conclusion, the final net effect is modestly beneficial for DBS on sleep disturbances, depending on other contributing factors.

Dopamine dysregulation syndrome (DDS) is characterized by excessive use of dopaminergic agents, beyond what is necessary for motor control. It is associated with severe LID and behavioral disturbances, including restlessness, aggression, dysphoria, anhedonia, and irritability during off periods, with compulsive demand for dopaminergic drugs. 83 The effects of STN or GPi DBS on DDS are still unclear and many groups have argued that STN DBS, in particular, may be beneficial by facilitating dose reduction of dopaminergic drugs. However, one recent study assessing the effect of STN and GPi DBS on DDS did not show any significant change; therefore, a neutral effect. 84 Moreover, a prior study had already shown mixed results with a significant proportion of patients showing improvement; however, most remained unchanged. 85 Finally, a larger series of patients who underwent STN DBS showed dramatic improvement. 86 In conclusion, the response of DDS to DBS (especially of the STN) is heterogeneous. Indication of this procedure in such cases must be cautious and done using a case-by-case approach depending on other aspects of behavioral and cognitive assessment.

### Features that may be potentially worsened by surgery

Impulse control disorders (ICDs) are compulsive disturbances commonly triggered by dopaminergic agents, especially dopamine agonists, which result in distress or impaired social and occupational functioning, commonly including pathological gambling, excessive shopping, and hypersexuality. 87 In general, ICDs can improve, worsen or even emerge after DBS, particularly of the STN. Despite the potential for improvement, the literature signals a cautious approach when considering surgery in these cases as the behavioral change may in fact be only a cursory manifestation of deeper and more widespread psychopathology. 88 Pooled data shows that screening for ICDs should be performed prior to DBS as a selection variable and that patients should be monitored for these problems during follow-up. 84

Speech is reported to worsen after surgery in more than half of all patients during the first year and almost all after the fifth year. 89 Occasionally, there has been improvement by STN DBS in speech intelligibility and articulation, however, these effects are transient and, in most instances, not clinically significant. Speech rate and rhythm are also affected, and stuttering can recur or be aggravated after STN DBS. These complications are less common for GPi DBS but delayed stimulation-induced dysarthria 5 to 6 years after surgery has also been described in these cases. 70

Cognitive deficits are consistently reported after STN DBS, especially regarding verbal fluency tasks. 89 Other cognitive domains are mildly but significantly affected, including memory, executive function, and abstract reasoning. These declines may be secondary not only to structural changes but also to the withdrawal of dopaminergic drugs known to interfere positively with cognitive performance in these areas. GPi DBS has a lower cognitive impact than STN procedures, with no significant effect six months after surgery, even in patients with advanced disease. 90 A meta-analysis of STN and GPi DBS reports over ten years concluded that cognitive and behavioral adverse events were more common in the STN than in the GPi group. 91 In well-selected patients, most studies have reported only mild or no significant deleterious effects of STN DBS in long-term neuropsychological performance, except for declines in verbal fluency tests. 84 There is a debate on whether cognitive disturbances may be more common with STN than GPi stimulation; however, results have suggested no significant difference.

Mood disorders (depression or mania), acute and transient or chronic and persistent, can occur in the postoperative period in STN DBS. 25 Additionally, suicidal tendencies have been reported in some patients with PD after STN DBS, with a suicide rate of 0.45% and an attempted suicide rate of 0.9%. 92 These rates were higher during the first year and associated with depression, being single, and previous history of ICDs or DDS. Various mechanisms might be involved in the pathophysiology of post-STN DBS depression, including reduction of dopaminergic drugs or indirect inhibition of the activity of ascending serotonergic neurons exerted by projections from the basal ganglia to the dorsal raphe nucleus. GPi DBS can also affect mood with transient but recurrent mania and hypomania described occasionally. 92 Recently the paradigm of suicide and suicidal ideation after DBS has been challenged by a study that analyzed 500 patients randomized for STN or GPi DBS and medical therapy. No cases of suicide or suicide attempt were detected. There were also no significant differences for any of the study arms concerning suicide ideation, which was rare (1.5% for STN DBS, 0.7% for GPi DBS, and 0.9% for medical treatment). In conclusion, due to these uncertainties, patients with unstable mood disorders should be considered at risk for postoperative worsening of psychiatric symptoms. All should be actively screened for such disorders before and after surgery.

In conclusion, for more than 30 years, DBS has emerged and evolved considerably and is now considered an effective therapeutic option for PD patients. The improvements observed after DBS in this setting have proved to be long-lasting and detectable even in more advanced stages. In this review, we discussed the available literature, providing a comprehensive overview of DBS therapy, including insights on therapeutic principles, adequate patient selection, precise timing of surgery, and well-established results in the short and long term.

As widely demonstrated, DBS improves the quality of life in PD patients who cannot be managed by medications alone and/or present complications due to levodopa therapy. As in any dynamic field, it is crucial for clinicians involved in this field to understand and remain updated on the evolution of DBS technology and the process of refinement and sophistication in terms of technical development, patient selection, and management. This review aimed to help fill in the knowledge gap, covering the fundamental aspects of DBS for the management of PD.
